# Healthcare Providers' Experiences With a Clinical Mentorship Intervention to Improve Reproductive, Maternal and Newborn Care in Mwanza, Tanzania

**DOI:** 10.3389/frhs.2022.792909

**Published:** 2022-05-06

**Authors:** Kahabi Isangula, Columba Mbekenga, Tumbwene Mwansisya, Loveluck Mwasha, Lucy Kisaka, Edna Selestine, David Siso, Thomas Rutachunzibwa, Secilia Mrema, Eunice Pallangyo

**Affiliations:** ^1^School of Nursing and Midwifery, Aga Khan University, Dar es Salaam, Tanzania; ^2^Aga Khan Health Services, Mwanza, Tanzania; ^3^Aga Khan Development Network, Dar es Salaam, Tanzania; ^4^Regional Medical Officer, Mwanza, Tanzania; ^5^Regional Reproductive and Child Health Coordinator, Mwanza, Tanzania

**Keywords:** clinical mentorship, on-the-job training, reproductive maternal and newborn health, Mwanza, Tanzania, rural, low-income countries

## Abstract

**Introduction:**

There is increasing evidence suggesting that clinical mentorship (CM) involving on-the-job training is one of the critical resources—friendly entry points for strengthening the knowledge and skills of healthcare providers (HCPs), which in turn facilitate the delivery of effective reproductive, maternal, and newborn health (RMNH) care. The article explores the experiences of HCPs following participation in the CM program for RMNH in eight districts of Mwanza Region in Tanzania.

**Materials and Methods:**

A qualitative descriptive design employing data from midterm project review meetings and Key Informant Interviews (KIIs) with purposefully selected HCPs (mentors and mentees) and District Medical Officers (DMOs) during endline evaluation were employed. Interview data were managed using Nvivo Software and analyzed thematically.

**Results:**

A total of 42 clinical mentors and master mentors responded to a questionnaire during the midterm review meeting. Then, a total of 17 KIIs were conducted with Mentees (8), Mentors (5), and DMOs (4) during endline evaluation. Five key themes emerged from participants' accounts: (i) the topics covered during CM visits; (ii) the benefits of CM; (iii) the challenges of CM; (iv) the drivers of CM sustainability; and (iv) suggestions for CM improvement. The topics of CM covered during visits included antenatal care, neonatal resuscitation, pregnancy monitoring, management of delivery complications, and infection control and prevention. The benefits of CM included increased knowledge, skills, confidence, and change in HCP's attitude and increased client service uptake, quality, and efficiency. The challenges of CM included inadequate equipment for learning and practice, the limited financial incentive to mentees, shortage of staff and time constraints, and weaker support from management. The drivers of CM sustainability included the willingness of mentees to continue with clinical practice, ongoing peer-to-peer mentorship, and integration of the mentorship program into district health plans. Finally, the suggestions for CM improvement included refresher training for mentors, engagement of more senior mentors, and extending mentorship beyond IMPACT catchment facilities.

**Conclusion:**

CM program appears to be a promising entry point to improving competence among HCPs and the quality and efficiency of RMNH services potentially contributing to the reduction of maternal and neonatal deaths. Addressing the challenges cited by participants, particularly the equipment for peer learning and practice, may increase the success of the CM program.

## Introduction

The past decade has seen increasingly rapid advancements and improvement in the field of reproductive, maternal, and newborn health (RMNH) care. However, Tanzania is among the countries that continue to document an unacceptably high maternal mortality ratio (556 per 100,000 live births) and neonatal mortality rate (26 per 1,000 live births) (1). Within Tanzania, Mwanza region has persistently reported higher RMNH indicators, most of which are above the national average. The region is part of the Lake Zone where the maternal mortality rate was 453 deaths per 100,000 live births and under-five mortality rate was 88 deaths per 1,000 live births (2). Neonatal mortality rate was also high (25 per 1,000 live births) in the 10-year period preceding the TDHS 2015/16 (1–3). Consequently, Mwanza is among the five prioritized underserved regions in Tanzania for targeted RMNH interventions by the government (2).

Under-investment in adequate education and training of healthcare providers (HCPs) is reported worldwide and contributes to the critical shortages, and lack of adequate and essential knowledge and skills (4–8). The mismatch between HCP educational strategies, health system requirements, and community demand directly affects the quality of RMNH, especially in the sub-Saharan African region (5). This calls for a change of strategy in formal educational training and continuous professional development to meet the critical requirement for skilled birth attendants that deliver a high-quality health services through the continuum of RMNH, especially in the underserved areas. Consequently, there is increasing demand for trainers to shift from traditional didactic training to innovative approaches that are more result oriented (6–11).

Recent evidence suggests that on-the-job training is one of the critical resources—friendly entry points for strengthening the knowledge and skills of HCPs, which in turn facilitate the delivery of effective RMNH interventions (6–10). On-the-job training through clinical mentorship (CM) of HCPs, coupled with health facility management mentoring, is considered to improve their clinical competencies in and performance of maternity and newborn care services (6–11). CM is a system of practical training and consultation that fosters ongoing professional development to yield sustainable high-quality clinical care outcomes (12). CM differs from conventional training because a clinical mentor is a healthcare provider (nurse/midwife or clinician) with substantial expertise in RMNH care who is trained on mentorship skills and can provide ongoing mentoring to less-experienced providers by reviewing clinical cases, assisting in case management, responding to questions, and providing feedback for practice improvement (12). The mentoring process can occur during facility visits and phone consultations. The WHO recognizes CM as critical to building successful networks of trained health care workers for RMNH and other conditions of public health interest in resource-constrained settings (12). It is within this context, the Improving Access to Reproductive, Maternal and Newborn Health in Mwanza, Tanzania (IMPACT) Project was implemented jointly by the Aga Khan Development Network and the Ministry of Health, Community Development, Gender, Elderly and Children in Tanzania (MoHCDEC) to improve RMNH. The details of the IMPACT project have been reported elsewhere (13, 14). However, following IMPACT training need assessment conducted at baseline, context-specific gaps in RMNH knowledge and skills among HCPs in the region were identified. In response, the IMPACT project and the School of Nursing and Midwifery at Agha Khan University in Tanzania designed and implemented a 1-year CM program for RMNH in Mwanza region.

The CM program aimed to improve the competencies of less experienced HCPs in relation to different aspects of RMNH that will ultimately improve the quality of care provided to the community in Mwanza. The main focus was on improvement in knowledge and skills in delivering different RMNH services and fostering positive attitudes toward RMNH clients as an entry point to quality care and better interpersonal relationships within RMNH care. This is because of a notable increase in client dissatisfaction with HCP's technical and interpersonal competencies within RMNH in recent years. Technical incompetence associated with skills, reliability, assurance, confidentiality, and patient engagement, and behavioral incompetence involving demeanor, attitudes empathy, communication skills/language, and respect continue to obscure the positive value of HCPs in the delivery of RMNH interventions in Tanzania [e.g.,(15, 16)].

The CM implementation protocol constituted a stepwise process that included (i) designing mentorship tools; (ii) identification and training of clinical and master mentors; (iii) pre-mentorship visits to healthcare facilities for identifying mentorship priorities and co-development of mentorship plans based on mentees' priorities; (iv) selection of appropriate mentorship approaches and methods; (v) conducting mentorship activities; (vi) conducting post-mentorship assessments and re-planning based on new needs of mentees; and (vii) mid-term progress review to address emerging gaps followed by an endline evaluation. Consequently, CM mentorship tools and training package were developed using the peer engagement process within Aga Khan entities drawing from current evidence (for instance, 6-12). The training package included theory and practical sessions on the following topics: the overview of the CM program, motivation in clinical mentorship, problem solving and decision making in health care (*Day 1*); interpersonal relationships in healthcare, communication skills in clinical mentorship, team building in clinical mentorship, and adult learning in clinical mentorship (*Day 2*); review of tools for clinical mentorship including clinical mentorship guide for mentors and master mentors (*Day 3*); piloting clinical mentorship tools in selected healthcare facilities in Sengerema district (*Day 4*); improvement of mentorship tools based on pilot exercise (*Day 5*); assignment of facilities of mentorship and development of mentorship action plan (*Day 6*) and finalization of logistics for mentorship visits (*Day 7)*. Then, a total of 35 clinical mentors and seven master mentors with exemplary clinical performance and knowledge were trained on mentorship skills for 1 week in October 2019. On the one hand, clinical mentors constituted HCPs identified from a list of the best performers in short courses that were conducted by AKU-SONAM under IMPACT prior to the implementation of CM intervention. Other criteria for selection of mentors included possessing good clinical skills, having significant experience in RMNH care, good work ethics, excellent communication skills, and good leadership skills. The selection of mentors ensured geographical representation to avoid long travels during the implementation of the program. The regional health management team and council health management teams were consulted to help identify mentors who met the selection criteria. On the other hand, master clinical mentors were the senior experts in RMNH who were charged with supporting clinical mentors in their mentorship endeavor as part of our attempt to create a supportive environment for continuous professional skills development among clinical mentors. This was a recognition that a 1-week training may not be sufficient enough to build desired clinical mentorship skills.

Trained clinical mentors were then offered letters of introduction to the managers of 80 health care facilities in eight districts of Mwanza Region, conducted a pre-mentorship visit to identify the mentorship needs of mentees in the 1–4 assigned facilities in early November 2019 and followed by monthly CM visits from late November 2019 to December 2020. The 80 healthcare facilities involved in CM intervention were those previously selected as the primary beneficiaries of the bigger IMPACT project (13, 14). During mentorship visits, clinical mentors conducted pre- and post-mentorship assessments to determine the immediate change in knowledge among mentees and developed a plan for the next mentorship sessions. Mentors were expected to consult with master mentors for technical support and/or joint mentorship as a way of improving their mentorship practices. The project coordination team facilitated mentors with clinical mentorship tools and a small stipend for meals and transport for every mentorship visit. KI provided overall technical leadership in the CM project implementation. A midterm implementation review meeting was conducted in June 2020 in Mwanza to assess progress, identify challenges, and generate solutions. An individual questionnaire was administered to all participants to capture their experience with the project at this stage. The project data indicated that by the end of CM program, 632 mentorship visits were conducted where 1,244 mentees were reached (67% women, 77% nurses, and 23% other cadres) and 38 master mentorship visits were conducted by senior HCPs to provide on-going capacity building on mentorship skills for mentors. Therefore, this study examined the HCP's accounts following participation in the CM intervention as part of on-the-job capacity building to deliver effective RMNH services in the Mwanza Region.

## Materials and Methods

### Design

The study employed a qualitative descriptive design (17) that facilitated tapping into HCPs' experiences of the CM program. A qualitative descriptive approach was deemed appropriate to answer the questions of *what* are the experiences of participation in the CM program, *what* challenges were encountered, and *how* can these challenges be addressed. The qualitative descriptive approach is appropriate to this inquiry as it seeks to develop an understanding and describe phenomena (e.g., experiences of participation in the CM program) without necessarily testing an existing theory (17). The approach offers an effective way of gathering a deeper and richer understanding of research participants' perceptions and perspectives. This takes into consideration that HCPs' experiences of CM program in our study context may differ from those of the providers in other contexts according to their expectations, resources, and relationships within healthcare settings. The qualitative descriptive design allowed us to acknowledge the subjectivity of experiences of both the participants and we, as researchers, to actively engage in the CM implementation and research process, and to collect data in a natural setting. Furthermore, by listening to participants' descriptions in Mwanza, the design allowed us to learn from the description of their experiences of CM and “…. using this knowledge to influence (future) interventions” and/or generate research findings of “specific relevance to practitioners and policy makers” in response to RMNH care [(17), p. 3]. This is in keeping with the broader aim of the CM program, which is to improve the competencies of less-experienced HCPs in relation to different aspects of RMNH that will ultimately improve the quality of care provided to the community in Mwanza and beyond.

### Settings

The CM intervention and the study were conducted in the Mwanza Region, which lies in the northern part of Tanzania, bordering Lake Victoria. The details of the Mwanza Region have been reported elsewhere (13, 14). Briefly, the 2017 Tanzania Human Development Report ranks the Mwanza region 13th among the 35 regions of Tanzania, with the population living in severe poverty (32.8%) and population vulnerable to poverty (19.7%) as compared to the national average of 31.3 and 18.2%, respectively (18). Health facility deliveries in the Mwanza Region account for 63.6% on average, while large disparities within the region persist with 87% of deliveries occurring in facilities in urban areas vs. 54.7% in rural areas (1, 2). Considering RMNH indicators, the Mwanza Region is one of five prioritized regions in Tanzania targeted by the Government to improve maternal and newborn healthcare (2). Within Mwanza, four districts (Ukerewe, Ilemela, Buchosa, and Magu) were purposefully selected based on core criteria to maximize the differentiation of factors that could affect practice and behaviors. These criteria were: (i) whether the district was on the mainland or an island; (ii) whether the district was Urban/Peri-Urban, or Rural; and (iii) approximate rate of delivery by a skilled health provider. Within each district, healthcare facilities that received clinical mentorship were purposefully selected.

### Sampling of Participants

All clinical mentors and master mentors were invited to fill out an individual questionnaire during the midterm review meeting. No specific selection criterion was applied other than being either a clinical mentor or a master mentor for CM intervention. Likewise, the qualitative component of the endline survey involved Key Informant Interviews (KIIs) with HCPs conducted as part of the endline survey using purposeful sampling. The inclusion criteria in KIIs were (i) any HCP who has received CM sessions from an IMPACT-trained mentor at his/her workstation during the past 6 months preceding the endline survey; (ii) any HCP who has delivered at least three peer-to-peer mentorship sessions to other HCPs in facilities other than their working station after receiving mentorship skills training from IMPACT; and (iii) a District Medical Officer (DMO) in a sampled district with firsthand knowledge about the CM program. No other criteria were applied during the participant's selection.

### Enrollment of Participants

During midterm review, all 35 clinical mentors and seven master mentors were invited to fill an individual questionnaire during the meeting as part of implementation progress monitoring. The meeting was conducted for 3 days in June 2020. During endline survey, the principles of data saturation guided the recruitment process for qualitative interviews. Recruiters were provided with the study selection criteria to guide the identification and invitation of HCPs and DMOs. Project implementation data were examined, a list of ~22 participants engaged in the CM intervention as mentors, mentees, and administrators were identified and their contacts were obtained. The initial communication was made by the IMPACT project implementation team with the help of the respective healthcare facility managers where the participants work. An incremental enrollment process was envisaged where additional participants were enrolled after the first few interviews until data saturation was reached. Data saturation was achieved after conducting 17 interviews. It is important to note that recent reviews have found that most qualitative studies arrive at data saturation after 9–17 interviews (19). Participants were allowed to choose a private room/office of their preference at the healthcare facility for KIIs. The use of both midterm review data and KIIs from endline survey as well as the inclusion of both HCPs and DMOs involved in the mentorship program facilitated data triangulation which is critical in ensuring qualitative research rigor (20).

### Data Collection Tools

A peer engagement strategy was employed to develop and translate both the questionnaire for clinical mentors and master mentors used during the midterm review and the semi-structured interview guides for KIIs for HCPs and DMOs used during endline survey into Swahili. Open-ended questions on the individual questionnaire for mid-term review ranged from topics of mentorship and challenges encountered to suggestions for improvements. Relatedly, an initial list of open-ended questions on the KIIs guides specifically focusing on CM was developed based on an overarching evaluation question: *How has IMPACT's health worker mentorship model contributed to strengthening RMNH health human resources in Mwanza?* The initial guides were subjected to review by experts within Aga Khan Development Network (Aga Khan University, Aga Khan Health Services, and Aga Khan Foundation). Topics for the final version of KII guide used for mentors and mentees ranged from how the CM model benefited the HCP and the facilities of mentorship, challenges encountered during CM visits to suggestions for improvement (HCP guide). Topics for the DMO guide ranged from improvements on RMNH in the district following implementation of the CM model and challenges of implementation to suggestions for improvement.

### Data Collection

As noted above, data used for this analysis were collected first, during the midterm project review in June 2020 conducted in Mwanza. About 42 self-administered individual questionnaires were distributed by the project staff to all 35 mentors and seven master mentors. All 42 questionnaires were returned at the end of the meeting, checked for completeness, and de-identified for analysis.

For KIIs conducted during endline evaluation, prior to data collection, six research assistants with bachelor's degree in social sciences and qualitative experience were recruited and trained by senior qualitative researcher for 5 days. The training focused on appropriate note taking, probing effectively, field reflection notes, ethical considerations, and informed consent. As part of the training, a pilot exercise involving two KIIs—one with male and one with female participants—was conducted by the research assistants in an urban facility to pre-test the interview guide in advance. The interview guides were then adjusted slightly to ensure suitability, comprehension by participants, length of discussion, and sensitivity. These two participants were excluded from subsequent interviews. All interviews were conducted in a convenient room/office. The room/office for the KIIs was confirmed with respondents in advance to enable respondents to identify an alternate location if required. Upon arrival, research assistants obtained informed consent and engaged respondents for ~45–60 min in a semi-structured dialog on the study content provided above (data collection tool). Each KII was conducted by one facilitator in a face-to-face encounter while adhering to COVID-19 preventive measures. All KII sessions were audio recorded, with the consent of participants, for translation and subsequent transcription. In addition, field notes were maintained to ensure all key findings and fieldwork descriptions are captured. The fieldwork team held debrief sessions daily after data collection to discuss how the sessions occurred and address any identified gaps or matters arising. The endline evaluation survey of the IMPACT project was conducted between September and October 2020.

### Data Management and Analysis

The individual questionnaires from the midterm review meeting were converted to electronic format, de-identified, and uploaded into Nvivo software for analysis together with KIIs transcripts. KII interview transcription and translation occurred simultaneously by research assistants and were verified by the research team. After transcription and translation, interview transcripts were de-identified, pseudonyms generated for each participant, and the data were uploaded into NVivo 11 software (QSR International, Australia) for deductive thematic coding.

Thematic analysis embraced Braun and Clarke's (21) approach and began after a couple of interviews and continued as more data were being gathered. More specifically, KI examined the research questions and interview guides and generated a list of initial themes and subthemes. These themes and subthemes were subjected to review by the research team members resulting in a consensual list. Then, KI coded the rest of the transcripts, refining and generating more codes upon coming across a new segment of data that could not fit into the initial codes. Codes were then sorted into existing subthemes and themes, followed by a collation of all relevant coded data extracts within identified themes. Throughout coding and refinement, peer consultation was maintained by frequently discussing within the research team to reflect on the themes and subthemes generated. Disagreements on the themes and subthemes were resolved through consensus building among research team members. Then, the themes and related contents were exported to an MS Word document for interpretation and report writing. Participants' accounts related to CM were used for this article.

### Ethical Considerations

The study received ethical clearance from the Aga Khan University ethics committee and the National Institute of Medical Research (NIMR/HQ/R.8a/Vol.IX/3444). Permission to conduct research was sought and received from local government authorities. Midterm review data were gathered as part of project implementation progress monitoring. During KIIs, all respondents provided verbal consent before participation. Respondents were invited to review the consent documents or have them read to them to ensure their full understanding. Those who agreed to participate were given a copy with study information and contact details to take home. All of the KIIs were held within an office/room in the healthcare facility selected by the respondent(s). All notes, transcripts, and audio recordings were kept in password-protected computers, stored temporarily in a secure room at the IMPACT office in Mwanza before transporting to a SONAM data center in Dar Es Salaam and were only accessed by the study team. All data used in reports were de-identified to ensure the anonymity of participants.

## Results

The midterm review questionnaire involved all 42 mentors and master mentors (60% women and 48% Nurses). Likewise, a total of 17 KIIs were conducted in four districts of the Mwanza Region. Participants included mentees (47%), mentors (29%), and DMOs (24%). Majority of participants were women (57%), nurses (47%), working at the dispensary (35%), and primarily performing clinical duties related to RMNH care (47%), with the 29% performing both clinical and administrative roles. Tables 1, 2 summarize participants' characteristics.

**Table 1:** Participants' demographics.

**Mid term review participants**				
	**Clinical mentors**	**Master mentors**		**Total**
**District**				
Ilemela	4 (11%)	2 (29%)		6 (13%)
Buchosa	4(11%)	0		4 (10%)
Magu	4(11%)	0		4(10%)
Ukerewe	4(11%)	0		4(10%)
Sengerema	5 (15%)	5 (71%)		10 (24%)
Nyamagana	8 (24%)	0		8 (18%)
Misungwi	4(11%)	0		4(10%)
Ngudu	2 (6%)	0		2(5%)
Total	35 (100%)	7 (100%)		42 (100%)
**Gender**				
Male	14 (40%)	3 (43%)		17 (40%)
Female	21 (60%)	4(57%)		25(60%)
Total	35 (100%)	7 (100%)		42 (100%)
**Cadre**				
Nurses and midwives	20 (57%)	4 (57%)		24 (57%)
Medical doctors	12 (34%)	3 (43%)		15 (36%)
Others (clinical officers, anaesthesiologists)	3 (9%)	0		3 (7%)
Total	35 (100%)	7 (100%)		42 (100%)
**Endline KII participants**				
	**Clinical mentors**	**DMOs**	**Mentees (HCPs)**	**Total**
**District**				
Ilemela	12	1	3	6 (35%)
Buchosa	0	1	1	2 (12%)
Magu	1	1	3	5 (29%)
Ukerewe	2	1	1	4 (24%)
Total	5 (29%)	4 (24%)	8 (47%)	17 (100%)
**Gender**				
Male	3	3	2	8 (47%)
Female	2	1	6	9 (53%)
Total	5	4	8	17 (100%)
**Cadre**				
Nurses	2	0	6	8 (47%)
Medical doctors	3	4	0	7 (41%)
Clinical officers	0	0	2	2(12%)
Total	5	4	8	17 (100%)
**Facility level**				
Hospital	2	4	3	5 (29%)
Health center	1	0	1	2 (12%)
Dispensary	2	0	4	6(35%)
Other (Admin)	4	0	0	4(24%)
Total	5	4	8	17 (100%)
**Primary responsibilities administrative**	0	4	0	4(24%)
Clinical (RMNH) only	3	0	5	8 (47%)
Both	2	0	3	5 (29%)
Total	5	4	8	17 (100%)

**Table 2:** KIIs participants' characteristics.

**Participant** ** category**	**Demographics**
DMOs	Male, medical doctor, overall in charge of the district health issues
	Male, Medical doctor, overall in charge of the district health issues
	Female, Medical doctor, overall in charge of the district health issues
	Male, Medical doctor, overall in charge of the district health issues
HCPs-Mentees	Female, Nurse, RCH incharge
	Male, Clinical Officer, Facility Incharge, Dispensary level
	Female, Nurse, works on MNH, Health center, Dispensary
	Female, Nurse, dispensary
	Male, Clinical officer, Dispensary
	Female, Nurse, District Hospital
	Female, Nurse, District Hospital
	Female, Nurse, District Hospital
HCPs-mentors	Male, Medical doctor, Regional Hospital
	Female, Nurse, Matron, Mentor, Dispensary
	Female, Medical Doctor, Facility incharge, Health center
	Male, Nurse, works at MCH, Dispensary
	Male, Medical doctor, District Hospital

### Summary of Key Findings

Clinical mentors and mentees cited the areas of on-the-job training as skills ranging from antenatal care, neonatal resuscitation, monitoring and management of complications related to pregnancy and delivery, to infection control and prevention. Clinical mentorship was cited to increase knowledge, skills, confidence, and change in the HCPs' attitude in RMNH service delivery on the one hand, and increased service uptake, quality, and efficiency and contributing to the reduction of maternal and neonatal mortality due to proper pregnancy and complication monitoring and timely response, on the other hand. The challenges of the mentorship program were inadequate equipment for learning and practice, the limited financial incentive to mentees, shortage of staff and associated time constraints, weaker support from management, and inadequate number of senior HCPs to conduct mentorship. The drivers of sustainability of the clinical mentor program and resulting changes were cited as the willingness of mentees to continue with the practice, ongoing peer-to-peer mentorship, the availability of necessary supportive structures, and the integration of the mentorship program into existing district health plans. Finally, the suggestions for improvement of mentorship program were refresher training for mentors, engagement of more senior mentors, and extending mentorship beyond IMPACT catchment facilities. Each of these issues are detailed below.

#### Areas of Capacity Building During the CM Program

**Figure 1:**
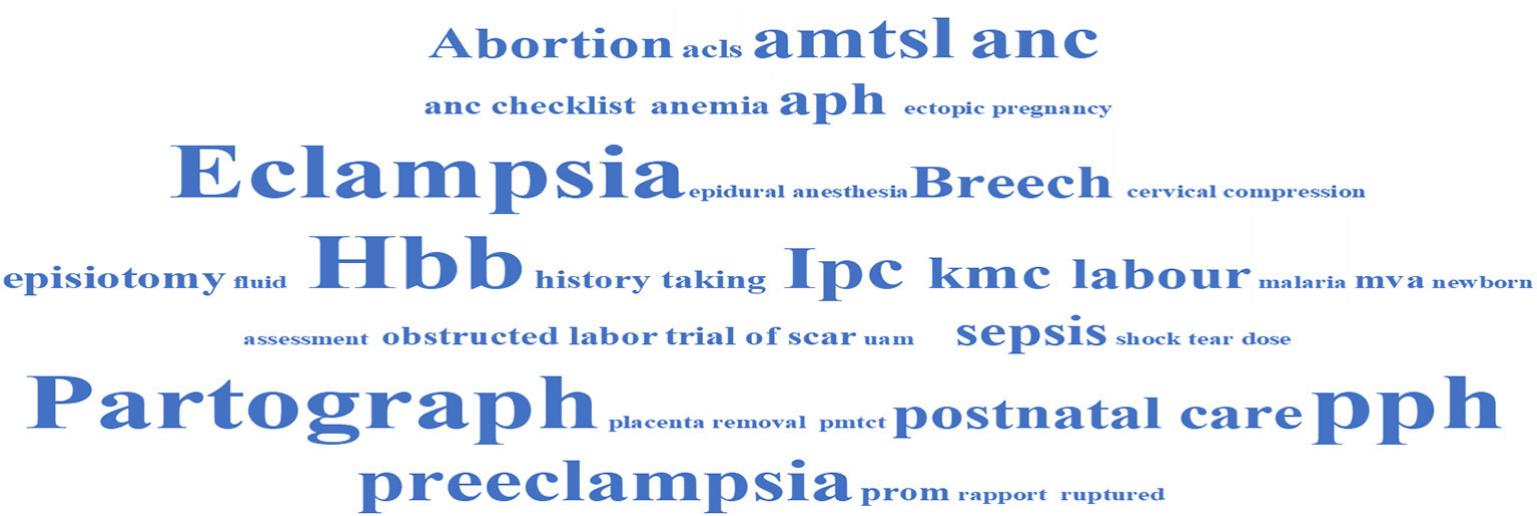
Common topics of clinical mentorship.

Participants were first asked about the areas of capacity building during the clinical mentorship program. From both the individual questionnaires administered during the midterm review meeting and KIIs conducted during the endline evaluation, several topics were reported by both mentors and mentored HCPs to have been implemented as part of the clinical mentorship to deliver RMNH services. Common topics cited included management of eclampsia and preeclampsia, neonatal resuscitation/Helping baby breathe (Hbb), filling and usage of partograph in monitoring progress of labor, management of post-partum hemorrhage (PPH), breech delivery, antenatal care (ANC), abortion care, active management of the third stage of labor (AMTSL), and infection prevention and control (IPC). Figure 1 summarizes the common topics of mentorship cited by participants.

#### Benefits of the CM Program

Respondents were asked about the usefulness of the CM model. The benefits of the mentorship model cited by participants of the midterm review and KIIs are those related to individual HCPs and those related to the improvement of RMNH services. Both individual and service improvement benefits were frequently cited together by participants. For instance, DMOs found the mentorship model useful to HCPs because it contributed to improved knowledge and skills among providers and also change their attitudes. Also, all DMOs affirmed that the CM program was practical oriented, cheap, and cost effective. These findings were echoed by the mentored HCPs who further reported the mentorship model to have increased their knowledge, skills, and confidence, and consequently improved the types and quality of RMNH services that they offered:

“*At a personal level, I have benefited much in that I can express myself at the theatre… that I can even stand and educate my peers irrespective of their age.... even in providing care to Mothers, my confidence has really increased from the mentorship I received.”* (Mentee, Nurse)

“*There are some changes that cannot be directly seen but I believe the mentorship has really helped like I said, to reduce maternal and infants' mortality. It might not be seen directly but the service providers' capacities have been improved.”* (DMO, Medical Doctor)

According to HCPs, the CM model facilitated gaining skills in the early detection of complications. CM was thus associated with more and better RMNH services. Similarly, DMOs and mentored HCPs said the mentorship model contributed to the reduction of complaints about the quality of services offered and contributed to the reduction of maternal and child deaths. The mentorship model was indeed associated with increased availability of quality RMNH services and reduced maternal and child deaths.

“*Since I mentored my colleagues, the major things that I have observed is that the death of infants has reduced; we have also been able to save so many women, who got PPH before or after delivery.”* (Mentor, Nurse)

“*I have received a lot of benefits. We have improved maternal and neonatal services. The services are now of good quality services and are provided on time*.” (Mentee, Nurse)

The benefits of CM were extended to increased ANC service uptake and responsiveness. Some HCPs suggested that mentorship increased the number of women who came to the clinics and who were served timely—and without the need to be referred to distant health facilities.

“*And …a very good example you may not believe it, Ukerewe is a district hospital that is separated by Lake Victoria and we have been going there to mentor them. The number of women who come to the clinics every month has increased and the clients who needed to cross (the lake to other facilities) for further examination and treatment can now get the services at the very same place that they are supposed to. All this was made possible because of that mentorship.”* (Mentor, medical doctor)

In summary, the CM was cited to have benefited the HCPs in terms of improving their knowledge, skills, confidence, and change in their attitude toward RMNH service delivery. The CM was also linked to increased service uptake, quality, and efficiency, and reduced maternal and neonatal mortality. Table 3 summarizes the benefits of CM cited by participants.

**Table 3:** Benefits and challenges of clinical mentorship.

**Benefits of clinical mentorship**		
**Benefits to individual HCPs**	**Benefits for RMNH services**	**Cross-cutting benefits**
• Increased knowledge and skills • Increased confidence • Facilitated change in attitudes • Increased HCP performance	• Improved quality, efficiency and timeliness of RMNH services offered • Increased availability of services • Increase in women using the clinic • Reduced maternal and child deaths	• Clinical mentorship program was regarded as practical-oriented, cheap and cost effective
**Challenges of clinical mentorship**		
**Challenges related to HCPs**	**Challenges related to CM program design**	**Challenges related to health service organization/management**
• Absenteeism while on leave • Opposition from colleagues • Non-adherence to the guidelines	• Inadequate equipment for mentorship sessions, peer learning and self-practice • Inadequate participation of senior HCPs trained as mentors	• Staff turnover due to transfers • Shortage of staff • Weaker support from local management • Time constraints

#### Challenges Encountered During the Implementation of the CM Program

The challenges encountered during the implementation of CM cited during midterm review and KIIs were 5-fold. First, inadequate equipment (e.g., models) for practical-based mentorship sessions. Inadequate equipment appeared to limit the learning experience during and after mentorship sessions where self-practice and peer learning are envisaged.

“*Another challenge is that we do not have the equipment to use for mentorship trainings so it becomes difficult to practice on our own when the mentor leaves.”* (Mentee, Clinical Officer)

Second, limited financial incentives for mentees. While there seemed to be a dedicated budget that was well-funded to support mentors' stipends in conventional training and mentorship visits, such support was cited as absent during subsequent mentorship sessions where mentees are not offered any financial incentives. This suggests that the notion of on-the-job training may not be well-conceived by some mentored HCPs.

“*Another challenge is when you go for regular trainings, there is some money that is given to participants. But when you come to mentor others, there is no money to give to participants.”* (Mentor, Nurse)

Third, human resources issues such as staff shortage and associated time constraints were cited as crippling the mentorship program. There was a need for RMNH care provision to continue undisturbed even as some providers worked as mentors or engaged as mentees. It was thus unsurprising to record the absenteeism of staff who had been scheduled as mentees. Furthermore, other challenges faced during mentorship as per HCPs included non-adherence to the guidelines, limited or delayed support from authorities, and opposition from colleagues. Oppositions from colleagues appear to be rooted in the prevailing inferiority–superiority complex in the healthcare sector and financial interests.

“*You may visit a facility and you find that providers show less interest or express uncertainties of your ability to mentor them based on your cadre” (Mentor, Nurse)*

“*The common challenges that we faced is the opposition that we sometimes receive from colleagues. Some may say, ‘you went for the program for all those weeks and you got good money, and now you are coming back just to educate us (without giving us some)’ (*Mentor, Nurse)

Another human resource challenge cited is staff turnover due to transfers. HCPs who received mentorship would be moved to other distant facilities and new HCPs would be posted where a mentorship session on a particular topic was recently completed. It was also common for mentors to be moved to distant facilities leaving their primary facility of mentorship unattended. This required program coordinators, mentors, and mentees to reorganize their resources, schedules, and topics in an attempt to address the challenge. The transfers also meant that the mentees did not get enough time to apply what they learned and observe change.

“*The problem comes when a staff is mentored on maternal and child health, then they get transferred to a facility that is not under the program, then those who are untrained are transferred to the facility under the program. Sometimes you meet with say 3 new faces transferred from another facility then you start mentorship a fresh because, those you had mentored and had started improving services at the facility have been transferred already.”* (Mentor, Medical doctor)

Fourth, weaker support from local management is marked by the bureaucratic process when seeking permission from supervisors to leave mentors' workstations to travel to far-off health facilities to mentor providers.

“*Challenges are many, even being accepted by leaders in a particular region… and you need to seek permission from the authority. Sometimes we are few and we weigh up the time taken seeking permission and we end up just breaching it and we are left to offer mentorship in fear of what will happen next...”* (Mentor, Clinical Officer)

And finally, inadequate senior HCPs to continue doing mentorship programs as per DMO's accounts. It is important to note that most senior HCPs who were trained as mentors did not subsequently conduct mentorship visits to their assigned facilities due to concerns of inadequate financial incentives.

“*There are challenges, you can never miss challenges …You know it is being done by IMPACT and that is why we see it as it is. You see, I do not have senior medical officers, I also lack senior nursing officers who are supposed to do these mentorships, because duties of graduate nurse and doctor is teaching, and we do not have them. You see, that is a very big challenge. We are even scared that the day IMPACT withdraws their staff who are supporting us – ‘How will we take off?’ and that is our biggest challenge*.” (DMO, Medical Doctor)

#### Sustainability of the CM Program and Resulting Changes

Participants of the midterm review meeting and KIIs were also asked about the sustainability of the mentorship program. The drivers of sustainability of the mentorship program were cited as 3-fold. First, the willingness of mentees to continue offering services because of the obvious benefits that mentorship has brought such as the availability of more and better services.

“*Clients are getting better services…. The skills that we have gained, we will continue to apply them so that, whenever you come back, you will find that we are still offering good services.”* (Mentor, Nurse)

Second, peer-to-peer mentorship because providers had been well-mentored. However, some HCPs suggested that sustenance of mentorship would require attention to necessary support and willingness of mentors and mentored HCPs to share and exchange the new knowledge and skills. HCPs who received training on mentorship skills were encouraged to keep on mentoring others. The availability of a mentor at a facility emerged as one of the most important contributors to the sustainability of mentorship because of other HCPs being able to reach out for information/assistance whenever in need:

“*In our facility, we have on-job training and what makes us happy is that one of our staff is a mentor... we also teach one another and remind one another…so we have to teach our colleagues. When you forget you ask for assistance.”* (Mentee, Nurse)

Third, the availability of necessary structures. HCPs said people were likely to continue with the CM program so long as there was a willingness to continue with the programs. Furthermore, HCPs noted that already the necessary structures such as guidelines and follow-up mechanisms were in place and they could also consider using whatever was readily available.

“*These mentorship and changes will continue because we have been enhanced in many areas. The facility now has equipment; it also has guidelines. When IMPACT leaves they will not take away the guidelines. They will leave us with the guidelines for ANC and family planning. And the equipment are there… We have given feedback; we have action plans that are in the files. We also have meetings where we will keep reminding each other. We have meetings for those managing data. When someone comes with the data indicating a drop in delivery, for example, if there were 14 deliveries and now, they are 4, we will have to follow up and know what caused it. We will address the concerns that are there. The methods that we used to motivate health workers to encourage the community to come and access services are the same ones we will use. So, they will continue ….” (Mentee, Clinical Officer)*

“*My opinion is that that service should continue and because IMPACT has really helped us, it is my belief that as a ministry, we should ensure it is sustainable. Because at the end of the day when a project comes to an end it goes with everything, it is because we do not absorb what has been brought to us… So, it is up to us the service providers to change that mindset that ‘The project is for IMPACT’... As service providers, we should absorb the knowledge were offered so that when they leave, we keep doing the good things that they have left with us.”* (DMO, medical Officer)

Fourth and finally, integration of the mentorship program into existing district health plans and use of IMPACT trained mentors for ongoing internal mentorships. DMOs affirmed that they have integrated mentorship into district budgets, are using and motivating trained mentors for internal mentorships, and are now training more senior mentors.

“*We have also incorporated (mentorship) in our budgets, so we do it internally … by using those who were trained by IMPACT, motivating them to continue mentorship. So, you find that there is a budget for professional development.”* (DMO, Medical Doctor)

“*Right now, we are training doctors and the strategy is that we want to have enough senior medical officers. Once we have senior medical officers as mentors the rest will fall into place so that these mentors are not lost*.” (DMO, Medical doctor)

#### Suggestions for Improvement of the CM Program

During the midterm review meeting and KIIs, HCPs, master mentors, and DMOs reported several overlapping areas for improvement of the mentorship model. There was a consensus among many participants on the need to ensure continuity of the mentorship program including refresher training—and consider having a trainer of trainers in every facility. This was cited to ensure providers got new updates and that incoming new staff got the necessary skills. Refresher training was also important because it provides an opportunity for providers to freshen knowledge acquired earlier.

“*I think the mentorship program should be continuous. This is because we keep having new updates all the time. When it comes to service providers, we always have new service providers employed. Therefore, it should be continuous because you can also forget at times. Some people are lazy in reading the guidelines. Therefore, the mentorship should be continuous.”* (Mentee, Nurse)

“*If the donors are still there then we should continue to get refresher trainings because things change every time, so any new knowledge that comes in, we should be informed.”* (Mentor, Nurse)

Some participants recommended having senior mentors (specialists) conducting mentorship in peripheral facilities because by having them, local community members were unlikely to need referrals and travel to distant health facilities—on poor road networks.

“*It will be good to have senior mentors in peripheral facilities. Specialists are still very few. Infrastructure, roads - some of the roads to those clinics are impassable. Some facilities do not have ambulances, so you find patients are carried using stretchers which take a long time to reach to hospital.”* (Mentor, Nurse)

Finally, some participants recommended scaling up and extending the clinical mentorship program beyond the IMPACT program catchment areas. This is in recognition that CM intervention was only implemented in 80 healthcare facilities that were the primary beneficiaries of the IMPACT project.

“*Mentorship should not be done only in the facilities under the [IMPACT] program rather, they should extend the mentorship to other facilities that are not under the program because maternal and child mortality does not occur only in facilities under the program but in all facilities.”* (Mentee, Nurse)

## Discussion

This study aimed at exploring the experiences of HCPs following the implementation of the CM program for RMNH services in the Mwanza Region. To achieve this objective, the study used the findings from two events, namely, the midterm project review questionnaire involving trained clinical mentors and master mentors and the KIIs with mentors, mentees, and district medical officers conducted at the endline to explore issues related to topics covered, benefits experienced, and challenges encountered during the implementation of the CM project, issues related to sustainability and suggestions for improvement of the program whenever the implementation of a similar program is envisaged in similar or different settings.

The findings of this study suggest a range of topics covered during the implementation of CM. These included management of eclampsia and preeclampsia, neonatal resuscitation, filling and usage of partograph in monitoring the progress of labor, management of post-partum hemorrhage, breach delivery, antenatal care, abortion care, active management of the third stage of labor, and infection prevention and control. Broadly, these topics mirror the priority capacity-building areas identified during a baseline study conducted before the commencement of the IMPACT project implementation. Likewise, most of the topics reflect the leading causes of maternal mortality and morbidity in Tanzania and have been prioritized both locally and globally. In Tanzania, these topics are prioritized in local RMNH manuals and guidelines, for instance, the Essential Newborn Care Guideline, the Basic Emergency Obstetric & Newborn Care Training manual, and the Comprehensive Emergency Obstetric and Newborn Care training manuals, just to cite a few (22–24). These topics have also been prioritized in CM intervention (6–11, 25) and suggested by the WHO as key capacity-building areas for improving maternal and newborn service delivery in healthcare facilities (26). Moreover, recent studies in the country have identified some of these topics as priority areas that need capacity building. For instance, a study examining the provision of Emergency Obstetric Care in Lake and Western zone regions including Mwanza where the present CM intervention was implemented (27) indicated weak implementation of signal functions such as newborn resuscitation and active management of the third stage of labor with weaker professional development as among the contributors. This implies that continued capacity building in these topics through in-service mentorship does not only reduce the skills gap among HCPs but also has the potential to contribute toward the reduction of maternal and newborn deaths in resource-constrained settings.

One of the important findings of this study are the experienced benefits of the CM program. These benefits emerged as being accrued to the providers themselves as well as RMNH services. CM emerged to have benefited the HCPs by improving their knowledge, skills, confidence, and change in their attitude toward the RMNH service delivery. The findings also indicate that CM contributed to increased service uptake, quality and efficiency, and contributed to the reduction of maternal and neonatal mortality. What these findings point to is the suggestion that the CM program achieved its objectives. As noted above, the CM project aimed to improve competencies, namely, knowledge, skills, and attitude among HCPs in relation to different aspects of RMNH that will ultimately improve the quality of care provided to the community in Mwanza. Furthermore, the findings strongly mirror the benefits of on-the-job/in-service training programs reported in previous studies elsewhere (6–11, 27, 28). For instance, a recent qualitative study involving HCPs in rural Tanzania has emphasized on-site mentoring and coaching visits as important entry points for quality improvement in maternal and child health services in the country (28). This suggests that CM programs have the potential to contribute to improved maternal and newborn services by cost-effectively addressing providers' competence gaps as they are delivered at the provider's workplace. This may explain why CM was further regarded as practical oriented, cheap, and cost-effective in the present study. Despite a mention of these cost-related benefits, establishing the cost-effectiveness of this CM intervention was beyond the focus of the present study.

Implementation of on-the-job training interventions is not without challenges. The findings unmasked many challenges, including inadequate equipment (e.g., models) for practical-based mentorship sessions. Inadequate equipment emerged to limit the learning experience during and after mentorship sessions where self-practice and peer learning are envisaged. This suggests that CM program designers need to ensure the availability of learning equipment for effective practical-based mentorship sessions, self-practice, and peer learning. The findings further unmasked concerns of limited financial incentives for mentees such as individual-level stipends during mentorship sessions. Financial incentives have not been widely described as a challenge in the implementation of the CM program in other areas, for instance, in Uganda (6) and Kenya (8). The call for financial incentives from mentees in Mwanza may imply that notion of on-the job-training may not be well-conceived by some HCPs. This suggests that more information needs to be provided to the mentees on the purpose and structure of on-the-job training to manage financial expectations from beneficiaries. Likewise, designing mentorship sessions to account for continuous professional development points may cushion against financial interests.

The findings further unmasked challenges related to a human resource such as shortage of staff and associated time constraints. These challenges emerged to cripple the mentorship program for there was a need for RMNH care provision to continue undisturbed even as some providers worked as mentors or engaged as mentees. Absenteeism, non-adherence to the guidelines, limited or delayed support from authorities, and opposition from colleagues also emerged. The oppositions encountered by mentors from some colleagues appear to be rooted in the prevailing inferiority–superiority complex in healthcare sector and financial interests (20). Addressing this inferiority–superiority complex may require prolonged behavioral change interventions that seeks to build willingness to learn from others and promote teamwork in healthcare settings.

There were concerns of staff turnover due to transfers by moving those who received mentorship to other distant facilities and new HCPs where a mentorship session on a particular topic was recently completed or has not been conducted. It was also common for mentors to be moved to distant facilities leaving their primary facility of mentorship unattended. COVID-19 further strained the human resource for health necessitating some trained mentors to be moved to the frontline. This negatively impacted mentorship visits for several months and it required program coordinators, mentors, and mentees to reorganize their resources, schedules, and topics in an attempt to address the challenge. The transfers also meant the trained mentees did not get enough time to apply what they learned and observe change. While the transfer of HCPs may be unavoidable due to ongoing demand and prioritization in healthcare service delivery, it is important to ensure that such endeavors do not create skills and competence deficits in facilities where providers are moved from. Continuous mentorship of remaining and new staff may somewhat cushion against transfer challenges.

The findings also indicated the inadequate willingness of senior HCPs to participate in CM sessions. Senior HCPs who were trained as mentors did not subsequently conduct mentorship visits to their assigned facilities. Non-engagement of senior HCPs in mentorship has been documented as a challenge in previous studies. For instance, a study in Uganda noted limited participation of medical doctors in the mentorship sessions as a challenge (6). In the present program, non-participation of senior HCPs was strongly linked to concerns about inadequate financial incentives. However, pre-occupation with other clinical and administrative responsibilities may have conflicted with their mentorship roles given their scarcity in the region and country (29, 30). This suggests that there is a need to either apply a clear selection criterion based on a voluntary spirit built on one's desire to serve lives first or address the financial incentive package for senior HCPs.

The findings unmasked the drivers of sustainability of the CM program including the willingness of mentees to continue offering services because of obvious benefits that mentorship has brought such as availability of more and better services; peer-to-peer mentorship because providers had been well-mentored; availability of necessary structures such as guidelines; the integration of the mentorship program into existing district health plans; and use of IMPACT-trained mentors for ongoing internal mentorships within respective districts. The availability of structures such as guidelines was cited in the context where recent studies continue to indicate the shortage of such tools in primary healthcare settings in the country (29–31). This implies the continued need to ensure the availability of service delivery guidelines for a successful CM program. However, the readiness of districts to integrate CM into their budgets and absorption of trained mentors for internal mentorships emerged as one of the critical strategies for sustainability. Follow-up studies may be needed to examine the experiences of these mentors after absorption into the district's supportive mentorship activities.

To improve the CM program, the findings indicate the need for refresher trainings—and consider having a trainer of trainers in every facility. Refresher training emerged as important because it was considered an opportunity for providers to refresh knowledge acquired earlier. Relatedly, having a trainer of trainers at every facility was considered important to ensure providers got new updates and that incoming new staff got necessary skills timely. Furthermore, the need for senior mentors (specialists) conducting mentorship in peripheral facilities was heightened because by having them, local community members were unlikely to need referrals and travel to distant health facilities—on poor road networks. This further explains why some participants recommended scaling up and extending the clinical mentorship program beyond the IMPACT program catchment areas. The problem with senior mentors however is the unwillingness to engage in CM because of limited financial incentives. This means, as proposed above, voluntary spirit-based selection criteria or resource mobilization to accommodate their financial desires may be required to maximize their engagement in mentorship.

## Limitations

The CM program met its objective of improving competencies, namely, knowledge, skills, and attitude among HCPs in relation to different aspects of RMNH that will ultimately improve the quality of care provided to the community in Mwanza. However, the study is not without limitations. First, the project itself was a complex and ambitious endeavor conducted in multiple districts. Mentors were selected from diverse facilities in different geographically isolated districts which posed a challenge to centralized monitoring and supervision. Although equal geographical representation is not a focus of qualitative inquiries (20), the engagement of participants from a few districts may have limited exploration of the whole range of experiences and challenges that manifested in all districts throughout the project. This may have resulted in underrepresentation of participants' insights from particular districts whose challenges may differ from the study sites included. However, the inclusion of data from both the midterm project review that involved all clinical mentors and master mentors aimed to partly address this limitation. Second, KII data collection occurred as part of an ambitious endline survey that included multiple teams working on different objectives. During an endline survey, a qualitative team that gathered data on the CM program was also responsible for gathering data on other objectives. Although engagement of mentors, mentees, and DMOs who were nurses, medical doctors, and clinical officers and inclusion of midterm review data may be a strength in terms of trustworthiness of the research through triangulation of multiple sources (20), collecting data as part of a much bigger endline study may be a limitation as it may have limited the prolonged engagement with the participants on the CM-specific aspect impacting on data richness. Third, the study largely relied on mentors, mentees, and the administrator (beneficiaries) to understand the experiences of CM implementation. While the accounts of these primary beneficiaries provided an insight into what they experienced, the experiences of project coordinators (specifically the School of Nursing at the Aga Khan University) and RMNH clients' perspectives (particularly women attending antenatal and postnatal clinics) on the quality of services post-mentorship program were not examined. Relatedly, the project sought to reinforce the skills, practice, and attitudes of providers. While we presented issues related to the improvement of skills and practices, issues related to improvement in HCPs' attitudes were not detailed enough in the data. We are of the view that such improvement in attitudes after CM intervention could better be examined from the perspectives of RMNH clients who received care from mentored HCPs. Future studies should therefore seek to engage coordinators to understand the challenges they face during project implementation and, RMNH clients to understand their experience and perspectives on the quality of technical and interpersonal services provided by mentees who received clinical mentorship. Finally, it is important to mention that the IMPACT project involved supply-side interventions, for example, health infrastructure improvement and human resource capacity building as well as supply side interventions, for example, use of community health workers and community sensitization for RMNH (13, 14). Members of the research team from the School of Nursing and Midwifery were only responsible for the human resource capacity-building component hence a focus on CM intervention. We acknowledge that there may be many contextual issues that continue to impact the actions of HCPs and experiences of clients within RMNH care, for instance, health service organization, accessibility, resources, and polices (27–31), consequently impacting how CM intervention is experienced by mentors and mentees as well as clients of mentored HCPs. Despite the limitations, we strongly believe the evidence generated in this study will form the basis for designing effective practical CM interventions and policy tools with a focus on addressing the issues that HCPs faced during CM implementation consequently improving their competence and contributing toward the improvement of RMNH indicators.

## Conclusions

The CM program was largely very well-received during its first at-scale implementation in the Mwanza Region. The program appears to be a promising entry point to improving competence among HCPs and the quality and efficiency of RMNH services, potentially reducing maternal and neonatal deaths. However, several challenges related to HCPs themselves, CM program design and health service organization/management were experienced in the course of CM implementation. Yet, mentees appear to portray a willingness to continue to apply the CM skills in RMNH service improvement, mentors are willing to continue to provide peer-to-peer mentorships and districts are willing to absorb trained mentors into ongoing supportive supervision and mentorship after the project. Therefore, addressing the challenges cited by participants, particularly the equipment for peer learning and practice and financial incentives, may increase the success of the CM program.

## Data Availability Statement

The raw data supporting the conclusions of this article will be made available by the authors, without undue reservation.

## Ethics Statement

The studies involving human participants were reviewed and approved by Aga Khan University Ethics Committee and the National Institute of Medical Research (NIMR/HQ/R.8a/Vol.IX/3444). Written informed consent for participation was not required for this study in accordance with the national legislation and the institutional requirements.

## Author Contributions

KI participated in the CM project design, coordination and monitoring of implementation, data collection, data analysis, and developing the manuscript. CM participated in the CM project conception and design, overall coordination and monitoring of the implementation, critically reviewed, and provided inputs to the manuscript. ES provided technical guidance in the manuscript development and commented on the manuscript. TM, LM, LK, ES, DS, TR, and SM commented on the final manuscript. All authors contributed to the article and approved the submitted version.

## Funding

This study received financial support from the Government of Canada and Aga Khan Foundation Canada as part of the IMPACT project.

## Conflict of Interest

The authors declare that the research was conducted in the absence of any commercial or financial relationships that could be construed as a potential conflict of interest.

## Publisher's Note

All claims expressed in this article are solely those of the authors and do not necessarily represent those of their affiliated organizations, or those of the publisher, the editors and the reviewers. Any product that may be evaluated in this article, or claim that may be made by its manufacturer, is not guaranteed or endorsed by the publisher.
