# Supplementary material for: Healthcare Providers' Experiences With a Clinical Mentorship Intervention to Improve Reproductive, Maternal and Newborn Care in Mwanza, Tanzania
Source: Front Health Serv. 2022 May 6;2:792909. doi: 10.3389/frhs.2022.792909 (PMC10012706; doi:10.3389/frhs.2022.792909)
Supplement: Supplementary file 1 [file Data_Sheet_1.PDF]

APPENDIX 1: DATA COLLECTION TOOLS

PART 1: INDIVIDUAL QUESTIONNAIRE DURING MID TERM REVIEW

DEMOGRAPHIC INFORMATION

- Mentor' name:
- District:
- Gender:
- Cadre:
- Facilities of mentorship:
- Date of filling the questionnaire

QUESTIONS

1. What are your experiences of participation in the CM intervention? (*Explain: important events during mentorship? What happened?)*

   ----

2. What are the areas that you provided mentorship the most? Why?

   ----

3. What changes have you noticed in RMNH services because of your mentorship? (*Explain: Changes among mentees, changes at the facility in general)*

   ----

4. What are your views on the support received from master mentors?

   ----

5. What support did you receive from master mentors and how useful was it?

   ----

6. What are the challenges you encountered in provision of clinical mentorship? (Technical, institutional, administrative? Individual?)

   ----

7. What are your suggestions for improvement of clinical mentorship?

   ----

8. Any other comment in relation to clinical mentorships?

THANK YOU:

PART 2: KII WITH MENTORS, MENTEES AND DMOs DURING ENDLINE SURVEY

- Ensure availability of quit and safe venue
- Welcome the participant
- Read the consent form

Interviewer:

Starting time:

PARTICIPANT DEMOGRAPHIC INFORMATION

1. District:
2. Gender:
3. Cadre:
4. Facility level:
5. Participant category:

KII QUESTIONS

1. INTERVIEW GUIDE FOR MENTORS:
   a. What are your roles and responsibilities in relation to RMNH care?
   b. How did participation in mentorship impact your practices at your primary facility?
   c. Now that the intervention has ended, what are your overall experiences of participation in the CM as a mentor? (Explain: important events during mentorship? What happened?)
   d. What are the areas that you provided mentorship the most? Why?
   e. What RMNH changes have you noticed among mentees and facilities because of your mentorship? *(Probe: change among mentees, change at the facility, changes among clients?)*
   f. Why do you think the changes noted will be sustainable?
   g. What are the challenges you encountered in provision of mentorship? (*Probe: resources, support, practice*?)
   h. What are your suggestions for improvement of clinical mentorship if it has to be implemented elsewhere?
   i. Any other suggestions?

2. INTERVIEW GUIDE FOR MENTEES

a. What are your roles and responsibilities in relation to RMNH care?
b. How did the mentorship sessions your received impact your practices at your facility?
c. Now that the intervention has ended, what are your overall experiences of participation in the CM as a mentee? (Explain: important events during mentorship? What happened?)
d. What are the areas that you received mentorship the most? Why?
e. What changes have you noticed during mentorship? *(Probe: change among mentors, change at the facility, changes among clients?)*
f. Why do you think the changes noted will be sustainable?
g. What are the challenges you encountered during mentorship and application of the skills gained? (*Probe: resources, support, practice*?)
h. What are your suggestions for improvement of clinical mentorship if it has to be implemented elsewhere?
i. Any other suggestions?

3. INTERVIEW GUIDE FOR DMOs

a. What are your roles and responsibilities in relation to RMNH care?
b. How did the mentorship intervention impact RMNH services in your district?
c. Now that the intervention has ended, what are your overall experiences of CM intervention as an administrator? (Explain: important events during mentorship? What happened?)
d. What are the areas that facilities received mentorship the most? Why?
e. What changes have you noticed in your district during mentorship? (Probe: changes among mentors, change at the facility, changes among clients? Changes in RMNH outcomes in general?)
f. How will your district sustain these changes?
g. What are the challenges encountered during mentorship? (Probe: resources, support, practice?)
h. What are your suggestions for improvement of clinical mentorship if it has to be implemented elsewhere?
i. Any other suggestions?

THANK YOU End Time,_________________________
